# Goal-Directed Movement Enhances Body Representation Updating

**DOI:** 10.3389/fnhum.2016.00329

**Published:** 2016-06-28

**Authors:** Wen Wen, Katsutoshi Muramatsu, Shunsuke Hamasaki, Qi An, Hiroshi Yamakawa, Yusuke Tamura, Atsushi Yamashita, Hajime Asama

**Affiliations:** Department of Precision Engineering, University of TokyoTokyo, Japan

**Keywords:** rubber hand illusion, body representation, sense of agency, sense of ownership, proprioceptive drift, goal, intention

## Abstract

Body representation refers to perception, memory, and cognition related to the body and is updated continuously by sensory input. The present study examined the influence of goals on body representation updating with two experiments of the rubber hand paradigm. In the experiments, participants moved their hidden left hands forward and backward either in response to instruction to touch a virtual object or without any specific goal, while a virtual left hand was presented 250 mm above the real hand and moved in synchrony with the real hand. Participants then provided information concerning the perceived heights of their real left hands and rated their sense of agency and ownership of the virtual hand. Results of Experiment 1 showed that when participants moved their hands with the goal of touching a virtual object and received feedback indicating goal attainment, the perceived positions of their real hands shifted more toward that of the virtual hand relative to that in the condition without a goal, indicating that their body representations underwent greater modification. Furthermore, results of Experiment 2 showed that the effect of goal-directed movement occurred in the active condition, in which participants moved their own hands, but did not occur in the passive condition, in which participants’ hands were moved by the experimenter. Therefore, we concluded that the sense of agency probably contributed to the updating of body representation involving goal-directed movement.

## Introduction

People’s perceptions of their own bodies constitute a fundamental aspect of self-consciousness and have been discussed by numerous researchers in multiple fields such as psychology, neuroscience, and engineering. Mental representation of the body involves both percepts and abstract knowledge, and beliefs and cognition about the body. Two concepts are usually used to describe body representation: body image and body schema (Gallagher, 1986; Paillard, 1999; de Vignemont, 2010). Usually, body image refers to perceptual, cognitive, or emotional awareness of the body, while body schema is more holistic and operates in a non-conscious manner, for example, containing certain habitual postures and movements (Gallagher, 1986). However, the definition and usage of these concepts is often unclear and confusing. Further, a prior study clarified body representation as belonging to two classes: perceptual representation (what the body is felt to be like), and cognitive representation (what the body is believed to be like; Longo et al., 2010). According to Longo et al. (2010), perceptual body representation involves online updating of the mental body, including superficial percepts and percepts of body size, shape, and posture, and influences our perception of external objects and behaviors. In contrast, cognitive body representation refers to the cognition of the body as a physical object in the external world (Longo et al., 2010), and is considered to be constructed and updated at the cognitive level. For example, in phantom limb pain, patients who have lost a limb by accident or amputation still have vivid percepts of the missing limb but know that it does not exist. In this case, the perceptual body representation in the brain has not been updated, but the cognitive representation has been updated (Longo et al., 2010). In the present study, we focused on the online updating of perceptual body representations in Longo et al.’s (2010) definition.

The underlying process of online body representation updating can be examined using the *rubber hand illusion*. In the original rubber hand illusion, after a fake rubber hand is brushed synchronously with the individual’s hidden real hand for a certain period, the individual feels the touch of the viewed brush, as if the rubber hand had sensed the touch (Botvinick and Cohen, 1998). Further, in the rubber hand illusion, individuals also experience the rubber hand as belonging to themselves (i.e., sense of ownership), and show a distorted sense of position of their real hands drifting to the rubber hand (Botvinick and Cohen, 1998). The rubber hand illusion reflects an interaction between vision, touch, and proprioception (Botvinick and Cohen, 1998). When the tactile input from the real hand matches visual information for the rubber hand, a sense of ownership of the real hand is extended to the rubber hand, and the incorrect position of the fake hand is integrated into the individual’s body representation and influences proprioception. Proprioceptive drift has been used widely as a behavioral indicator of body representation updating in the rubber hand illusion (Botvinick and Cohen, 1998; Tsakiris and Haggard, 2005a; Tsakiris et al., 2006; Costantini and Haggard, 2007; Kalckert and Ehrsson, 2012). In addition, the extension of body ownership in the rubber hand illusion has been examined not only via subjective rating of ownership (Shimada et al., 2009; Guterstam et al., 2011; Kalckert and Ehrsson, 2012, 2014) but also via objective measures such as skin conductance responses (Armel and Ramachandran, 2003; Ehrsson et al., 2008) and muscle activity (Perez-Marcos et al., 2009; Slater et al., 2009; Moretto et al., 2011). Although previous studies suggested common mechanisms for proprioceptive drift and the illusory sense of ownership (Botvinick and Cohen, 1998; Kalckert and Ehrsson, 2012, 2014), recent findings reported a distinction between them, suggesting that different multisensory mechanisms underlie the two phenomena (Kammers et al., 2009; Rohde et al., 2011; Blanke, 2012). Blanke (2012) suggested that body consciousness involves three aspects: self-identification (i.e., body ownership), self-location (where “I” am in space), and first-person perspective (the experience of the position from where “I” perceive the world), and that these aspects are probably based on distinct brain mechanisms.

Tsakiris and Haggard (2005b) examined the role of body schema on the rubber hand illusion, and found that proprioceptive drift occurred only when the rubber hand’s posture was similar in appearance to that of the real hand. The results indicated that the bottom-up integration of multiple sensory input is necessary, but not sufficient for body representation updating, and the illusion is modulated by top-down influences originating from one’s body representation (Tsakiris and Haggard, 2005b). Moreover, a recent study using immersive virtual reality demonstrated that the illusion of ownership could be generated from the realistic appearance of a fake (virtual) body and first-person perspective, even without any other congruent visuotactile or sensorimotor cues (Maselli and Slater, 2013), revealing the strong influence of body schema and first-person perspective in the illusion. Recent research using visuo-motor adaptation showed that synchronous movements can also produce the rubber hand illusion (Tsakiris et al., 2006; Kalckert and Ehrsson, 2012, 2014). In such paradigms, both the sense of ownership (i.e., the feeling that the fake hand belongs to oneself; Gallagher, 2000) and the sense of agency (i.e., the feeling that the movements of the fake hand are generated by oneself; Gallagher, 2000) are generated for the rubber hand. In Tsakiris et al.’s (2006) experiment, the participants watched a projection of their own hands on a screen and moved their index or little fingers. When the finger movements were generated by external forces, proprioceptive drift occurred only for the moving finger; in contrast, when participants moved their fingers themselves, proprioceptive drift also occurred for unmoving fingers, indicating that motor agency integrated distinct body parts into a coherent and unified whole (Tsakiris et al., 2006). Moreover, Tsakiris et al. (2007) pointed out that although afferent signals alone could generate sense of ownership and modify body representation, a more coherent experience of the body depends on the integration of both effect and afferent signals in an action context.

In the present study, we aimed to examine the influence of goal-related instruction and goal-attainment feedback on body representation updating, using the visuo-motor adapted version of the rubber hand paradigm. Goal-directed movements are very common in daily life. Prior studies have reported that goals and goal-attainment feedback greatly influenced sense of agency during action (Aarts et al., 2005; Metcalfe and Greene, 2007; Metcalfe et al., 2013; van der Weiden et al., 2013; Wen et al., 2015b,c). Sense of agency could play an important role in integrating body representation during body movement (Tsakiris et al., 2006, 2007). In the present study, we hypothesized that goal-directed movements may improve body representation updating, and sense of agency may play a role in this process. This issue is important in understanding the processes underlying body representation and helps to clarify the internal relationship between different factors underlying self-consciousness including sense of agency, ownership, and body representation. In the present study, we first clarified the effect of goal-directed movement on body representation updating in Experiment 1 by examining whether proprioceptive drift differed between conditions with and without a specific goal or goal-attainment feedback. Thereafter, we further examined the role of sense of agency in Experiment 2, in which we compared the effect of goals on proprioceptive drift between the active and passive conditions.

## Experiment 1

### Methods

The present study used the rubber hand paradigm with virtual reality stimuli. The participant moved his or her hidden left hand forward and backward, while a virtual left hand was presented 250 mm above the real hand and moved in synchrony with it. Participants were instructed to move their hands according to cued timing (without a specific goal) or an instruction to touch a moving object (with a specific goal). We examined the effects of having a goal and goal-attainment feedback on proprioceptive drift. The study was approved by the ethics committee of the School of Engineering at the University of Tokyo, and written informed consent was obtained from all participants prior to participation.

#### Participants

Twenty students with normal or corrected-to-normal visual acuity participated in the experiment (three women, mean age = 22.8 years, *SD* = 2.0, range 20–25 years). The sample size was chosen because it provided power of 0.95 in revealing the difference between the conditions with and without a goal, based on the results for the first five samples (*a priori* power was computed using G*Power 3, Faul et al., 2007).

#### Stimuli and Tasks

**Figure 1:**
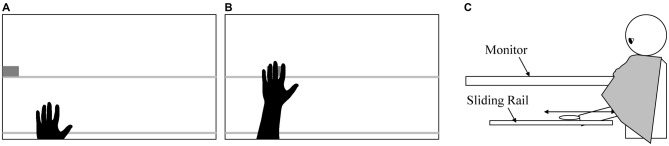
**Example of the screen that presented a virtual hand and flying object in the conditions with the goal involving touching an object (A), example of the screen showing the virtual hand touching the flying object (B) and arrangement of the experimental devices (C)**.

A virtual left hand was presented on the left side of a 597 × 336 mm (width × height) blue screen using OpenGL (an application programing interface for rendering graphics, Figures 1A,B). The size of the virtual hand was 105 × 145 mm (width × height), similar to the average size of a normal hand. The virtual hand moved between the bottom and center of the screen, in synchrony with the participant’s hand, and an arm was presented when the virtual hand moved to the center of the screen, giving the appearance of the virtual hand being stretched out from the bottom of the screen. Two horizontal lines were represented at the bottom and center of the screen, 149 mm apart. The color of the two lines alternated between red and white at random intervals of 4–6 s in the conditions without goals. Participants placed their hands on a sliding rail to restrict hand movements in a horizontal direction (Figure 1C). The distance between the sliding rail and the surface of the screen was 250 mm. A 3-D position sensor (PHANToM premium 1.5, Sensable Inc.) was attached to the participant’s index finger to measure hand position. The average delay in response in synchronous conditions was below 30 ms and was unperceivable for participants.

In each trial, participants placed their left hand on the sliding rail and moved it forward and backward according to the stimuli on the screen. They were instructed to move the hand forward until the root of the index finger reached the upper line on the screen (e.g., Figure 1B) and backward until the root of the thumb reached the lower line on the screen (e.g., Figure 1A). Participants were told that they were not strictly required to move the distance indicated, as it was for use as a reference, and they should move the hand while maintaining a comfortable posture. Participants were also told to keep the hand at the initial position (Figure 1A) between movements.

There were five conditions between trials (Table 1). In the random condition, participants moved the left hand upon the same instruction in the no-goal and delay conditions; however, the virtual hand moved forward and backward at random times without responding to the position of the participant’s hand (the number of times the virtual hand moved was equal to the number of cues). In the random condition, the rubber hand illusion was not considered to have occurred and responses served as a baseline for other conditions. In the no-goal and delay conditions, participants were told to move the left hand forward and backward when the lines changed color (every 4–6 s). They were told to complete this movement within approximately 1.5 s. After the movement, they rested the left hand on the rail to wait for the next cue. In the no-goal condition, the movement of the virtual hand responded to the position of the real hand immediately. In the delay condition, the virtual hand responded to the position of the real hand after a 1000 ms delay. We used the delay condition to manipulate agency on an intermediate level (Farrer et al., 2013; Wen et al., 2015a) to examine the hypothesis that agency could contribute to body representation updating.

**Table 1:** **List of conditions in the experimental task**.

	Synchronized stimuli	Goal involving touching an object	Goal-attainment feedback
Random	No	No	–
Delay	1000 ms delay	No	–
No goal	Yes	No	–
Goal without feedback	Yes	Yes	No
Goal with feedback	Yes	Yes	Yes

In the goal-without-feedback and goal-with-feedback conditions, the two lines on the screen remained white in color, and a red block emerged from the left of the screen and along the upper line at a speed of 100 mm/s, every 4–6 s. The participant was instructed to move his or her hand forward to touch the flying object with the virtual hand. The task was easy to achieve, and all participants were able to touch the flying object once they had practiced. For participants in the goal-without-feedback condition, the object continued to fly, even after being touched, but disappeared when it passed the center of the screen. In the goal-with-feedback condition, the object stopped after being touched and disappeared 1 s later. The object disappeared after passing the center of the screen if participants did not touch it. The five conditions are listed in Table 1. The number of cues (color changing of the lines, flying object) in each trial of each condition was equal (36 in each trial); therefore, the number of hand movements in each trial should have been the same when participants followed the instruction.

In each trial, participants performed hand movements for 3 min. Previous studies observed proprioceptive drift after a visuo-motor adaption for 1.5–3 min (Tsakiris et al., 2006; Kalckert and Ehrsson, 2012); therefore, we believe 3 min should be enough to observe the rubber hand illusion. Thereafter, participants pointed to the perceived height of the left hand on a ruler, which was placed to their right, using the right index finger with their eyes closed. We did not measure pre- or post-drift for each trial. Instead, proprioceptive drift in the random condition served as a baseline, and responses in other conditions were compared with the baseline. After the 3 min hand movement, participants verbally rated two statements that referred to the feeling of ownership (“I felt as if the virtual hand was my hand”) and agency (“the virtual hand moved just like I wanted it to, as if it was obeying my will”) on a 7-point Likert scale (−3: totally disagree; 0: uncertain; +3: totally agree). The two statements were modified from those used in prior studies examining the rubber hand illusion (Botvinick and Cohen, 1998; Kalckert and Ehrsson, 2012).

#### Procedure

Participants were tested individually in a quiet room, seated in front of a 27″ LCD monitor. The height of the chair was adjusted to ensure that participants’ eyes were approximately 350 mm above the surface of the monitor. Participants’ arms and the bottom of the monitor were covered with a blanket, to ensure that participants could not see the movement of their own arms. Prior to each trial, the initial position of the participant’s left hand was adjusted to ensure that the virtual hand appeared above the real hand. Each condition comprised three blocked trials, resulting in a total of 15 trials. Prior to each condition, participants received an explanation regarding the task and practiced the movement for 1 min. After each trial, participants detached the 3-D position sensor from the finger, exposed the hand, and took a 3 min break. The order of conditions was randomized between participants. The experiment lasted for approximately 110 min on average.

### Results

#### Proprioceptive Drift

**Figure 2:**
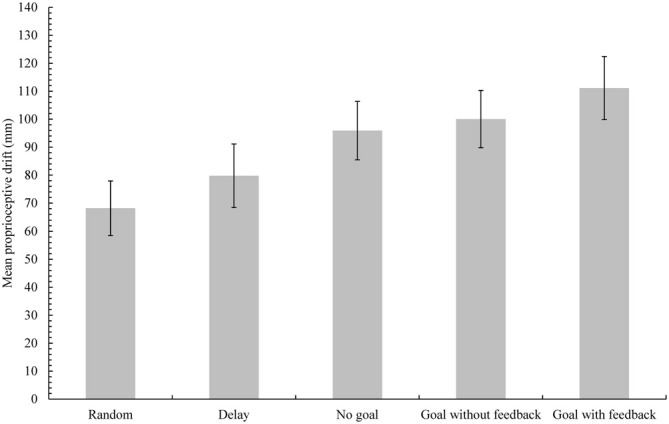
**Mean proprioceptive drift in each condition in Experiment 1.** Error bars represent standard errors. Proprioceptive drift in the goal-with-feedback condition differed significantly from that of the no-goal condition, but the difference between the goal-without-feedback and no-goal conditions was nonsignificant.

Average proprioceptive drift (difference from actual height) and standard errors for each condition are shown in Figure 2. We conducted a repeated measures analyses of variance (ANOVA) to examine the influence of condition on proprioceptive drift. The main effect of condition was significant (*F*_(4,76)_ = 23.58, *p* < 0.001, $\eta_{p}^{2}$ = 0.55; random: *M* = 68.18 mm, *SD* = 43.46 mm; delay: *M* = 79.77 mm, *SD* = 50.82 mm; no-goal: *M* = 95.92 mm, *SD* = 46.80 mm; goal-without-feedback: *M* = 100.03 mm, *SD* = 45.82 mm; goal-with-feedback: *M* = 111.13 mm, *SD* = 50.51 mm). For *post hoc* comparisons between conditions, the significance level was set at 0.005 according to the Bonferroni correction. Proprioceptive drift in the no-goal, goal-without-feedback, and goal-with-feedback conditions differed significantly from those in the random and delay conditions (random and no-goal conditions, *t*_(19)_ = −4.70, *p* < 0.001; random and goal-without-feedback conditions, *t*_(19)_ = −5.78, *p* < 0.001; random and goal-with-feedback conditions, *t*_(19)_ = −6.49, *p* < 0.001; delay and no-goal conditions, *t*_(19)_ = −4.34, *p* < 0.001; delay and goal-without-feedback conditions, *t*_(19)_ = −5.33, *p* < 0.001; delay and goal-with-feedback, *t*_(19)_ = −6.82, *p* < 0.001). More importantly, the proprioceptive drift in the goal-with-feedback condition was significantly larger relative to that observed in the no-goal condition (*t*_(19)_ = 3.91, *p* = 0.001) but did not differ from that observed in the goal-without-feedback condition (*t*_(19)_ = 2.82, *p* = 0.011). The difference between the no-goal and goal-without-feedback conditions was nonsignificant (*t*_(19)_ = −1.02, *p* = 0.320).

#### Ownership and Agency Ratings

**Figure 3:**
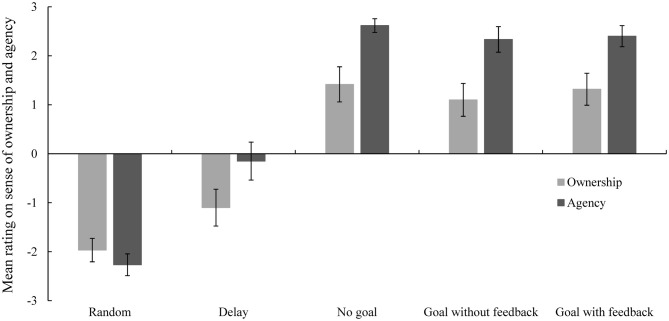
**Mean ratings for sense of ownership and agency in each condition in Experiment 1.** Error bars represent standard errors. Sense of ownership ratings in the conditions with synchronous movement of the virtual hand were significantly higher relative to those observed in the conditions in which movement of the virtual hand was delayed or completely random. The sense of agency rating in the delayed condition was significantly higher relative to that observed in the random condition, and the ratings in the conditions with synchronous movement of the virtual hand were significantly higher relative to those observed in the delayed and random conditions.

The means and standard errors for ownership and agency ratings for the virtual hand are presented in Figure 3. With respect to the sense of ownership rating, the repeated measures ANOVA revealed a significant main effect of condition (*F*_(4,76)_ = 53.93, *p* < 0.001, $\eta_{p}^{2}$ = 0.74; random: *M* = −1.97, *SD* = 1.07; delay: *M* = −1.10, *SD* = 1.68; no-goal: *M* = 1.42, *SD* = 1.60; goal-without-feedback: *M* = 1.10, *SD* = 1.50; goal-with-feedback: *M* = 1.32, *SD* = 1.46). A significance level of 0.005 was used for *post hoc* multiple comparisons according to the Bonferroni correction. Ratings in the no-goal, goal-without-feedback, and goal-with-feedback conditions were significantly higher relative to those observed in the random and delayed conditions (random and no-goal conditions, *t*_(19)_ = −9.77, *p* < 0.001; random and goal-without-feedback conditions, *t*_(19)_ = −9.39, *p* < 0.001; random and goal-with-feedback conditions, *t*_(19)_ = −10.14, *p* < 0.001; delay and no-goal conditions, *t*_(19)_ = −7.52, *p* < 0.001; delay and goal-without-feedback conditions, *t*_(19)_ = −6.26, *p* < 0.001; delay and goal-with-feedback, *t*_(19)_ = −7.21, *p* < 0.001). The differences between the random and delay conditions, and the differences between no-goal, goal-without-feedback, and goal-with-feedback conditions were nonsignificant (random and delay: *t*_(19)_ = −2.01, *p* = 0.059; no-goal and goal-without-feedback conditions: *t*_(19)_ = 2.65, *p* = 0.016; no-goal and goal-with-feedback conditions: *t*_(19)_ = 0.69, *p* = 0.500; and goal-without-feedback and goal-with-feedback conditions: *t*_(19)_ = −1.66, *p* = 0.114).

With respect to sense of agency ratings, the main effect of condition was significant (*F*_(4,76)_ = 114.39, *p* < 0.001, $\eta_{p}^{2}$ = 0.86; random: *M* = −2.27, *SD* = 0.99; delay: *M* = −0.15, *SD* = 1.73; no-goal: *M* = 2.62, *SD* = 0.62; goal-without-feedback: *M* = 2.33, *SD* = 1.16; goal-with-feedback: *M* = 2.40, *SD* = 0.96). A significance level of 0.005 was used for *post hoc* multiple comparisons according to the Bonferroni correction. The rating in the random condition was significantly lower relative to those observed in the other conditions (random and delay conditions, *t*_(19)_ = −4.47, *p* < 0.001; random and no-goal conditions, *t*_(19)_ = −20.80, *p* < 0.001; random and goal-without-feedback, *t*_(19)_ = −15.20, *p* < 0.001; random and goal-with-feedback, *t*_(19)_ = −17.62, *p* < 0.001). The rating in the delay condition was significantly lower relative to those observed in the no-goal, goal-without-feedback, and goal-with-feedback conditions (delay and no-goal conditions, *t*_(19)_ = −8.43, *p* < 0.001; delay and goal-without-feedback, *t*_(19)_ = −7.94, *p* < 0.001; delay and goal-with-feedback conditions, *t*_(19)_ = −8.78, *p* < 0.001). However, the differences between the no-goal, goal-without-feedback, and goal-with-feedback conditions were all nonsignificant (no-goal and goal-without-feedback conditions: *t*_(19)_ = 1.69, *p* = 0.108; no-goal and goal-with-feedback conditions: *t*_(19)_ = 1.49, *p* = 0.153; and goal-without-feedback and goal-with-feedback conditions: *t*_(19)_ = −0.456, *p* = 0.654).

### Discussion

The purpose of Experiment 1 was to examine the influence of goals and goal-attainment feedback on body representation updating, using the rubber hand paradigm with virtual reality stimuli. We found that when the participants moved their hands according to the instruction to touch a virtual object and received goal-attainment feedback, proprioceptive drift was larger relative to that observed when they moved without a specific goal and merely followed cued timings, indicating that goal-directed movement significantly enhanced body representation updating. In addition, when goal-attainment feedback was not provided, the instruction to touch a virtual object did not affect proprioceptive drift, indicating that congruence between goal and feedback was important in body representation updating.

The present study provided the first evidence indicating that goal-directed movement led to greater enhancement of body representation updating relative to that observed for movement without a specific goal. In the no-goal condition, the participants showed larger proprioceptive drifts relative to those in the random condition, indicating that changes in body representation occurred within a short period during the experimental task. Moreover, in the goal-with-feedback condition, proprioceptive drifts were significantly larger than those in the no-goal condition. When there was a specific goal, the expectation of goal achievement was probably generated on a high cognitive level, and when it was congruent with the goal-attainment feedback, this enhanced body representation updating, into which incorrect visual information was integrated to a greater extent.

Why would congruence between goals and goal-attainment feedback enhance interactions between motor control, vision, and proprioception in the rubber hand illusion? It is possible that sense of agency played an important role in this phenomenon. Prior research has shown that congruence between goals and goal-attainment feedback, besides that between motor commands and sensory feedback, exerted a strong influence on sense of agency (Metcalfe and Greene, 2007; Metcalfe et al., 2013; Wen et al., 2015b,c). Further, Tsakiris et al. (2006) found that sense of agency was important in integrating distinct body parts into coherent and unified bodily awareness. In addition, recent studies have shown that intention of action (without actual movement) can also produce a weaker form of the rubber hand illusion with a brain-computer-interface (Perez-Marcos et al., 2009; Slater et al., 2009), revealing the potential role of agency in construction of body representation. Therefore, goals and goal-attainment feedback probably enhanced body representation updating via sense of agency in our experiment. We tested this hypothesis further in Experiment 2, comparing the effects of goal-directed movement between the active and passive conditions. Prior research has shown that intention regarding action plays a critical role in sense of agency, and people experience a strong sense of agency only during active movement (Tsakiris et al., 2006; Kalckert and Ehrsson, 2012). If sense of agency plays an important role in the effects of goal-directed movement on body representation updating during active movement, this effect should be greater relative to that observed with passive movement.

## Experiment 2

### Methods

#### Participants

In total, 17 students with normal or corrected-to-normal visual acuity participated in the experiment (four women, mean age = 25.8 years, *SD* = 3.7, range 20–33 years). The participants were naïve to the purpose of the experiment and were different from the participants in Experiment 1. Results for one participant were excluded from the analysis because the 3-D position sensor was moved by mistake and resulted in inconsistent positioning for the real and virtual hands. *A priori* power analysis using G*Power 3 (Faul et al., 2007) based on the results of proprioceptive drifts in the no-goal and goal-with-feedback conditions in Experiment 1 showed that a sample size of 16 provided power of 0.95 in revealing the difference between the conditions with/without a goal.

#### Stimuli, Task, and Procedure

The experimental paradigm used in Experiment 1 was also used in Experiment 2. Participants moved their hands forward and backward on a sliding rail, which was placed under a table, while watching a virtual hand moving synchronously on a horizontally placed monitor (Figure 1). In contrast to Experiment 1, participants moved their hands actively (active condition) or passively (passive condition), with the goal of touching a virtual object (goal-directed condition) or in response to the colors of lines on the monitor (no-goal condition).

The goal-directed and no-goal conditions in Experiment 2 were the same as the goal-with-feedback and no-goal conditions, respectively, in Experiment 1. In the active condition, participants were instructed to move their hands actively (as in Experiment 1). In the passive condition, participants were told to place their relaxed left hands on the sliding rail. The experimenter moved the part of the sliding rail on which the participant’s hand was placed, using two ropes connected to the front and back. Participants were instructed to control their hand movements, even when they knew their hands would be moved. Each participant completed 12 trials, three for each goal condition (goal directed vs. no goal) and mode of movement (active vs. passive). The order of the goal conditions was randomized between participants. The mode of movement was blocked and the order counterbalanced between participants. Short breaks, during which participants released their hands from the device and exposed them to vision, were taken between trials. The experiment lasted approximately 60 min on average.

### Results

#### Proprioceptive Drift

**Figure 4:**
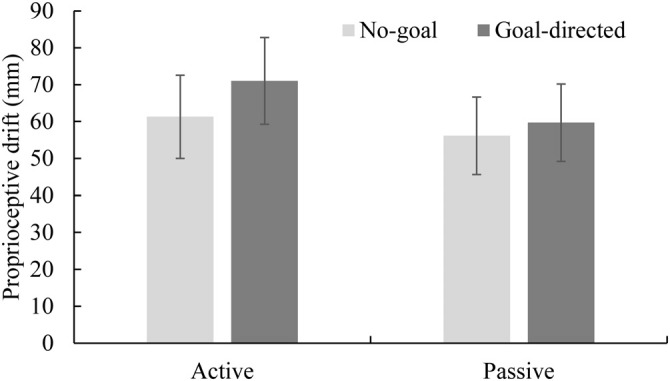
**Mean proprioceptive drift in each condition in Experiment 2.** Error bars represent standard errors. Proprioceptive drift in the goal-directed active condition differed significantly from that of the no-goal active condition.

Average proprioceptive drift (difference from actual height) and standard errors for each condition are shown in Figure 4. A 2 (movement: active or passive) × 2 (goal condition: goal directed or no goal) repeated measures ANOVA revealed a significant interaction between mode of movement and goal (*F*_(1,15)_ = 4.78, *p* = 0.045, $\eta_{p}^{2}$ = 0.24; no-goal active: *M* = 61.29 mm, *SD* = 45.14 mm; goal-directed active: *M* = 71.02 mm, *SD* = 47.05 mm; no-goal passive: *M* = 56.14 mm, *SD* = 41.99 mm; goal-directed passive: *M* = 59.70 mm, *SD* = 41.97 mm). The main effects of goal condition and mode of movement were nonsignificant (*F*_(1,15)_ = 4.51, *p* = 0.051, $\eta_{p}^{2}$ = 0.23 and *F*_(1,15)_ = 3.31, *p* = 0.089, $\eta_{p}^{2}$ = 0.18, respectively). Because our interest was the effect of goal in both the active and passive conditions, we conducted comparisons between the goal directed and no goal conditions in the two movement conditions. The proprioceptive drift in the goal-directed condition was greater relative to that observed in the no-goal condition only when participants moved their hands themselves (active condition, *t*_(15)_ = 2.51, *p* = 0.024). The goal conditions did not differ significantly when participants’ hands were moved by the experimenter (passive condition, *t*_(15)_ = 1.22, *p* = 0.240).

#### Ownership and Agency Ratings

**Figure 5:**
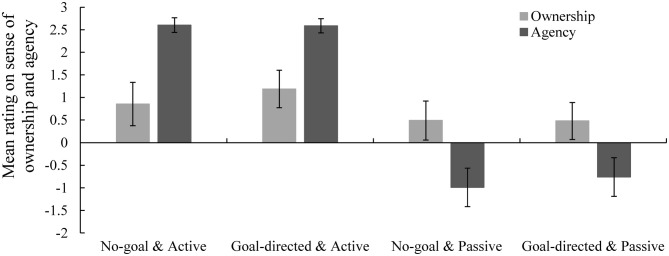
**Mean ratings for sense of ownership and agency in each condition in Experiment 2.** Error bars represent standard errors. Sense of ownership ratings did not differ between the active and passive or no-goal and goal-directed conditions. Sense of agency ratings differed significantly between the active and passive conditions but did not differ between the no-goal and goal-directed conditions.

The means and standard errors for ownership and agency ratings for the virtual hand are presented in Figure 5. With respect to the sense of ownership rating, ANOVA results showed that the main effects of mode of movement and goal and the interaction between them were nonsignificant (*F*_(1,15)_ = 2.63, *p* = 0.126, $\eta_{p}^{2}$ = 0.15; *F*_(1,15)_ = 2.88, *p* = 0.110, $\eta_{p}^{2}$ = 0.16; and *F*_(1,15)_ = 3.74, *p* = 0.072, $\eta_{p}^{2}$ = 0.20, respectively; no-goal active: *M* = 0.85; *SD* = 1.92; goal-directed active: *M* = 1.19; *SD* = 1.66; no-goal passive: *M* = 0.49; *SD* = 1.72; goal-directed passive: *M* = 0.48; *SD* = 1.63). With respect to the sense of agency rating, the main effect of mode of movement was significant (*F*_(1,15)_ = 62.26, *p* < 0.001, $\eta_{p}^{2}$ = 0.81), showing that the manipulation of agency was successful. The main effect of goal condition and the interaction between mode of movement and goal condition were nonsignificant (*F*_(1,15)_ = 1.09, *p* = 0.314, $\eta_{p}^{2}$ = 0.07 and *F*_(1,15)_ = 1.72, *p* = 0.209, $\eta_{p}^{2}$ = 0.10, respectively; no-goal active: *M* = 2.60; *SD* = 0.65; goal-directed active: *M* = 2.59; *SD* = 0.63; no-goal passive: *M* = −0.99; *SD* = 1.70; goal-directed passive: *M* = −0.76; *SD* = 1.71).

### Discussion

In this experiment, we compared the effect of having a goal on proprioceptive drift in active and passive movement. In the active condition, the participants reported a strong sense of agency regarding the movement of the virtual hand, and proprioceptive drift in the goal-directed condition was greater relative to that observed in the no-goal condition. In contrast, in the passive condition, the participants reported a poor sense of agency regarding the virtual hand, and the effect of having a goal on proprioceptive drift was no longer present. Therefore, we concluded that sense of agency probably played an important role in the effect of goal-directed movement on body representation updating, as we hypothesized in the Discussion section for Experiment 1.

## General Discussion

In the present study, we examined the influence of having a goal and goal-attainment feedback on proprioceptive drift, as an index of body representation updating in the rubber hand illusion. The results of Experiment 1 showed that the proprioceptive drift observed when participants intentionally moved their hands to achieve a specific goal and received goal-attainment feedback was significantly greater relative that observed when they moved their hands according to cued timing without any specific goal. In addition, the intention to achieve a goal did not affect proprioception without goal-attainment feedback, indicating that the comparison of goal-achievement intention and goal-attainment feedback is critical. Furthermore, to examine our hypothesis that the sense of agency would play an important role in this phenomenon, we compared the effect of having a goal on proprioceptive drift between the active and passive conditions in Experiment 2. The results clearly supported our hypothesis, showing that having a goal enhanced proprioceptive drift only in the active condition, in which participants reported a strong sense of agency regarding the virtual hand, but it had no effect in the passive condition, in which the participants did not experience a sense of agency (or felt a very weak sense of agency).

This study was the first to report that having a goal enhanced body representation updating (as discussed in Experiment 1). Further, it was also the first study to provide evidence indicating that sense of agency played a critical role in this phenomenon. Previous studies have reported that high-level cognitive processes, including the comparison of goals and goal attainment, greatly influence sense of agency (Metcalfe and Greene, 2007; Metcalfe et al., 2013; van der Weiden et al., 2013; Wen et al., 2015b,c, 2016). Additionally, knowing the goal of others’ actions induces illusionary sense of agency, even when one’s own body remains static (Wegner et al., 2004). However, no studies have been conducted to determine whether these processes also influence body representation updating, while sense of agency is closely connected with body representation and probably contributes to the integration of distinct body parts into a coherent whole (Tsakiris et al., 2006, 2007). However, we do not suggest that sense of agency directly enhances body representation updating, as there was no difference in proprioceptive drift between the active and passive condition when there was no goal (Experiment 2). The results of the present study showed that high-level cognitive processes involved in comparing goal-achievement intention with goal-attainment feedback contributed to body representation updating, and sense of agency was required in this process. In contrast, processes in body representation updating based on the congruency of multiple (low-level) sensory input probably do not require sense of agency.

With respect to intentions during body movement, a previous study categorized them into three types: distal (intention to achieve an overarching goal), proximal (decision to start acting now), and motor (intention to recall a motor representation; Pacherie, 2008). The goal in the present study involved distal intention and was activated only in the condition involving the goal of touching the virtual object. Apraxia patients provide evidence of a distinction between distal and motor intention. Apraxia usually occurs because of damage to the left frontoparietal cortex (Buxbaum et al., 2003; Pazzaglia and Galli, 2014) and involves impairment in performing planned/purposeful movement but not in the motor representation itself (Leiguarda and Marsden, 2000). Further, sense of agency is usually impaired in apraxia, suggesting a functional link between sense of agency and distal intention and a structural link between agency and apraxia (de Jong, 2011; Pazzaglia and Galli, 2014). Moreover, apraxia patients also experience difficulty in evoking and representing conceptual knowledge regarding the human body (Goldenberg, 1995; Buxbaum et al., 2000), suggesting a link between distal intention (goal-directed action) and body representation construction/updating. In the present study, when participants had a goal, distal intention was activated and probably contributed to the generation of body consciousness, enhancing body representation updating. Conscious intention is a key factor in sense of agency (Haggard, 2005), and distal intentions were reported to increase the subjective sense of agency (Vinding et al., 2013). We suggest that intention is also a key factor in the observed effect of goal-directed movements in body representation updating. More important, we found that intention alone was not enough. Proprioceptive drift was enhanced only when there was feedback on goal attainment in addition to the goal, indicating that the reconstructive processes involving effects of actions were also important in body representation updating.

Further, in our experiments, we did not observe effects of having a goal on subjective ratings of agency and ownership. Agency and ownership ratings in the rubber hand paradigm are usually taken as explicit indices of the illusion, while proprioceptive drift is taken as an implicit index of body ownership. However, we suggest that proprioceptive drift reflects body representation, while agency and ownership ratings reflect the extension of body consciousness, instead of body representation itself. In other words, agency and ownership ratings refer to subjective feelings about external stimuli (e.g., the virtual hand) rather than one’s own body. The discrimination between body ownership and proprioception was also reported in previous behavior studies (Kammers et al., 2009; Rohde et al., 2011) and supported by neuroscience research (Blanke, 2012). Further, a prior study reported that people’s ratings were higher for ownership in a passive-movement condition compared to an active-movement condition, indicating that extension of body ownership mainly relies on congruence between vision and tactile or proprioceptive cues (Walsh et al., 2011). In contrast, we suggest that updating of body representation may comprise multi-level processes, including both sensory and high-level cognitive processes. Additionally, in the present study, we used only two statements in the subjective rating of the illusion, which was much fewer than previous studies (Botvinick and Cohen, 1998; Kalckert and Ehrsson, 2012, 2014). This might have weakened the precision in measurement of ownership and agency. Nevertheless, the relationship between different aspects of body consciousness deserves further examination in future research.

In conclusion, using the rubber hand paradigm of visuo-motor adaption, we found that when people received goal-attainment feedback, goal-directed movement enhanced proprioceptive drift to a greater extent relative to that observed in the condition without a specific goal. We also found that the effect of having a goal occurred in active, but not passive, movement, demonstrating the important role of sense of agency in this phenomenon. The findings of the present study could provide useful knowledge for practical applications involving body representation. For example, immersive virtual reality involving limb movements has been used to treat phantom limb pain (Murray et al., 2007), as it temporally solved the inconsistencies between body representation and disabled limbs and facilitates body representation updating in the brain. According to the results of the present study, adding an overarching goal to limb movements or enhancing sense of agency for such movements could improve treatment effectiveness.

## Author Contributions

Conceived and designed the experiments: WW, KM, SH, QA, HY, YT, AY, HA. Performed the experiments: WW, KM. Analyzed the data: WW, KM. Contributed reagents/materials/analysis tools: WW, KM. Wrote the article: WW.

## Conflict of Interest Statement

The authors declare that the research was conducted in the absence of any commercial or financial relationships that could be construed as a potential conflict of interest.
